# Intermolecular Interactions of Edaravone in Aqueous Solutions of Ethaline and Glyceline Inferred from Experiments and Quantum Chemistry Computations

**DOI:** 10.3390/molecules28020629

**Published:** 2023-01-07

**Authors:** Piotr Cysewski, Tomasz Jeliński, Maciej Przybyłek

**Affiliations:** Department of Physical Chemistry, Pharmacy Faculty, Collegium Medicum of Bydgoszcz, Nicolaus Copernicus University in Toruń, Kurpińskiego 5, 85-096 Bydgoszcz, Poland

**Keywords:** edaravone, tautomers, ethaline, glyceline, solubility, wet eutectic solvents, COSMO-RS, intermolecular interactions, linear regression

## Abstract

Edaravone, acting as a cerebral protective agent, is administered to treat acute brain infarction. Its poor solubility is addressed here by means of optimizing the composition of the aqueous choline chloride (ChCl)-based eutectic solvents prepared with ethylene glycol (EG) or glycerol (GL) in the three different designed solvents compositions. The slurry method was used for spectroscopic solubility determination in temperatures between 298.15 K and 313.15 K. Measurements confirmed that ethaline (ETA = ChCl:EG = 1:2) and glyceline (GLE = ChCl:GL = 1:2) are very effective solvents for edaravone. The solubility at 298.15 K in the optimal compositions was found to be equal x_E_ = 0.158 (c_E_ = 302.96 mg/mL) and x_E_ = 0.105 (c_E_ = 191.06 mg/mL) for glyceline and ethaline, respectively. In addition, it was documented that wetting of neat eutectic mixtures increases edaravone solubility which is a fortunate circumstance not only from the perspective of a solubility advantage but also addresses high hygroscopicity of eutectic mixtures. The aqueous mixture with 0.6 mole fraction of the optimal composition yielded solubility values at 298.15 K equal to x_E_ = 0.193 (c_E_ = 459.69 mg/mL) and x_E_ = 0.145 (c_E_ = 344.22 mg/mL) for glyceline and ethaline, respectively. Since GLE is a pharmaceutically acceptable solvent, it is possible to consider this as a potential new liquid form of this drug with a tunable dosage. In fact, the recommended amount of edaravone administered to patients can be easily achieved using the studied systems. The observed high solubility is interpreted in terms of intermolecular interactions computed using the Conductor-like Screening Model for Real Solvents (COSMO-RS) approach and corrected for accounting of electron correlation, zero-point vibrational energy and basis set superposition errors. Extensive conformational search allowed for identifying the most probable contacts, the thermodynamic and geometric features of which were collected and discussed. It was documented that edaravone can form stable dimers stabilized via stacking interactions between five-membered heterocyclic rings. In addition, edaravone can act as a hydrogen bond acceptor with all components of the studied systems with the highest affinities to ion pairs of ETA and GLE. Finally, the linear regression model was formulated, which can accurately estimate edaravone solubility utilizing molecular descriptors obtained from COSMO-RS computations. This enables the screening of new eutectic solvents for finding greener replacers of designed solvents. The theoretical analysis of tautomeric equilibria confirmed that keto-isomer edaravone is predominant in the bulk liquid phase of all considered deep eutectic solvents (DES).

## 1. Introduction

Solubility is one of the most basic properties, which is of a particular importance in the case of pharmaceutically active ingredients (APIs). The studies on solubility including both equilibrium and kinetic approaches are involved in several important areas such as liquid dosage improvement [1], bioavailability enhancement [2], crystallization [3], preformulation [4] and thermodynamic modelling [5,6].

Edaravone (5-methyl-2-phenyl-4H-pyrazol-3-one, CAS RN [89-25-8]), also known as Radicava, is used for ischemic stroke [7,8] and amyotrophic lateral sclerosis (ALS) treatment [7,9]. These neuroprotection applications are related to the anti-oxidant activity of edaravone, which is an effective free radical scavenger [10,11]. The Biopharmaceutics Classification System (BCS) categorizes Edaravone as a class IV drug seriously suffering from poor aqueous solubility which results in low bioavailability and prevents from oral pharmaceutical formulations. That is why many attempts were made to study edaravone solubility in various neat and multi-component solvents. For example, the solubility of this drug was examined in binary aqueous solvents containing methanol, ethanol and isopropanol [12,13], in different organic/organic solvents comprising ethyl acetate, acetonitrile, methanol, ethanol, n-propanol [12,13,14] and in such neat solvents as toluene [14], acetone [14,15], isopropanol [14], 1,4-dioxane, acetonitrile, dichloromethane, ethyl acetate, methanol, ethanol, n-propanol, isopropanol, n-butanol, sec-butanol, isobutanol, n-pentanol and water [15]. According to the study of Li et al. [14], when considering only neat solvents, the edaravone solubility increases accordingly: acetonitrile < toluene < acetone < methanol < ethanol < isopropanol < ethyl acetate < ethanol < n-propanol. As it can be inferred from this comparison, the edaravone solubilization prediction is not a trivial task, since there is no clear correlation with the solvent polarity or hydrogen bond accepting/donating preferences. The probable reason for this is the complex behavior of edaravone in solvents due to tautomeric equilibria. This issue was raised both experimentally and theoretically [16,17,18]. It was argued [16] that in chloroform or acetonitrile solutions only the keto-tautomer is measurable as inferred from observed ^13^C-NMR spectra. In other solvents, including methanol, pyridine, DMSO and trifluoroethanol, a mixture of the edaravone tautomers is detectable with solvent-dependent populations. Additionally, using the Abraham descriptors for assessments of the proportions of tautomers in various solvents, Liu et al. [19] also concluded that solvents significantly influence the predominant form of edaravone. This is important, since the keto-form is characterized by significantly less hydrogen bond donating properties compared to enol and amine isomers, which affects interactions with active proton-containing groups of solvents.

Ethaline (ETA) and glyceline (GLE) are popular deep eutectic solvents (DES), which are interesting due to their unique properties and environmental friendliness, which were applied in extraction or extractive distillation processes [20,21], absorption refrigeration systems [22,23], greenhouse gases capture (CO_2,_ CH_4_) [24,25] and as lubricants [26,27]. The basic physicochemical properties of ETA and GLE were studied in detail using various experimental methods [28,29,30]. As it was established by Mjalli and Ahmed [29], density, viscosity, refractive index, pH and conductivity are significantly affected by temperature and composition. The ETA and GLE properties are attributed to the intermolecular interactions between the components forming the DES mixture. Moreover, the addition of a third component, such as urea, significantly disturbs the network of interactions in DES, as evidenced by the significant changes in melting points in the case of ETA-urea systems compared to pure ETA mixtures [31]. The results of molecular dynamics studies reported by Celebi et al. [32] indicate that in the ETA aqueous mixtures, when the water mass fraction is 40%, several types of complexes involving choline cation (Ch^+^) can be distinguished, namely Ch^+^-ethylene glycol, Ch^+^-Cl^−^ and Ch^+^-Ch^+^. Of course, the latter complex is relatively unstable. In addition to electrostatic repulsion, the factor that additionally affects the weakening of this ephemeral complex is the solvation of Ch^+^ ions [32]. The significant influence of the composition of the mixture and temperature on the variability of dipole–dipole interactions is the likely cause of the ultrasonic velocity changes observed by Jabbar et al. [33] during studies conducted for various DES in water, including ETA. The spectroscopic studies of Kalhor et al. [34] showed that, after mixing of DES consisting of choline chloride and ethylene glycol in the molar proportion of 1:2 with D_2_O, the solvation leads to the Cl^−^⋯H-O interaction cleavage in choline chloride. Further, the addition of D_2_O leads to the formation of ethylene glycol dimers and trimers. On the other hand, the studies on the surface tension of mixtures containing ETA and water showed that choline cation and ethylene glycol form intermolecular interactions in the diluted ethaline/air interface layer [35]. As can be seen from the above-mentioned examples, the composition and addition of water have a significant effect on the structural features and nature of the interactions occurring in ETA and GLE. This is also reflected in the complex phase behavior and specific thermophysical properties [36,37,38,39].

In general, DES are considered as promising green solvents. The main advantages of DES are low volatility [40], biodegradability [41,42], safety and low toxicity compared to conventional solvents and sometimes ionic liquids [43,44]. However, there are also critical opinions regarding their safety and toxicity [45]. Solvents play an important role in the pharmaceutical industry. It is estimated that they account for 80–90% of the total amount of chemicals used in drug manufacturing [46]. For this reason, the selection of environmentally friendly solvents that can be used in the pharmaceutical industry is a crucial issue. Apart from the environmental friendliness, the solubility of pharmaceuticals is the key property, which should be taken into account at the solvent selection stage of the process design.

The aim of this study was to optimize the designed solvents’ composition for increasing the solubility of edaravone. In particular, saturated systems in ETA, GLE and their aqueous mixtures were studied experimentally and rationalized using the results of quantum chemistry computations. Finally, the predictive model was formulated utilizing COSMO-RS molecular descriptors for the development of a multiple linear regression (MLR) model.

## 2. Results and Discussion

This work addresses the poor solubility of edaravone both from experimental and theoretical perspectives. Although many organic solvents were used [12,13,14,15] for the characteristics of edaravone saturated condition, there are still two main issues with such a collection. First of all, the utilized solvents can hardly be considered green. Secondly, the amount of edaravone dissolution in ambient conditions is at most moderate. This paper proposes an alternative perspective by focusing on solubility in non-volatile eutectic solvents mixed with water.

### 2.1. Experimental Solubility Determination of Edaravone in DES

As already mentioned, various systems have been studied in the context of edaravone solubility, since its poor water solubility is known to be a serious disadvantage. Here, instead of organic solvent mixtures, ethaline (ETA) and glyceline (GLE), two very popular eutectic solvents, were used. Here, the DES comprising choline chloride (ChCL) with either glycerol (GL) or ethylene glycol (EG) was studied. The selection of such DES constituents is based on the observation that natural deep eutectic solvents NADES are more effective solvent if choline chloride is mixed with polyalcohols with a lower number of hydroxyl groups. Hence, applying ethylene glycol as a replacement for glycerol seems to be promising in terms of increasing the solubility of active pharmaceutical ingredients, although it is hardly considered a green solvent [41]. However, ethaline [47,48,49] and glyceline [50,51,52] were quite often used as designed solvents for a variety of purposes.

During the first stage of experiments, three different compositions of DES were tested. This included a unimolar proportion of both constituents, as well as a 2 and 4 times excess amount of either glycerol or ethylene glycol. No compositions with an excess amount of choline chloride were used, again based on earlier experiences. The solubility of edaravone in these systems was determined in the temperature range from 298.15 K to 313.15 K with 5 K intervals and the results were collected in Table 1. When inspecting the obtained results, three observations are evident. First of all, the increase in temperature results in elevated solubility of edaravone. When comparing the mole fraction values at 298.15 K and 313.15 K, it turns out that the mole fraction solubility has increased by about 3 times in the case of DES systems containing glycerol and about 2.5 times in the case of ethylene glycol. There was no significant difference in terms of temperature-dependent solubility increase between the studied molar ratios, although for both systems this increase was the lowest in the case of the 1:2 ratio. Secondly, the composition involving a 2-times excess amount of the polyolic DES constituent contributes to the highest solubility in the case of both systems. It yielded the highest amounts of dissolved edaravone in all temperatures with the 1:4 system coming second in terms of solubility and the 1:1 composition, which is characterized by the lowest solubility compared to the previously mentioned solvents. At 298.15 K, the solubility of edaravone in the best performing composition was x_E_ = 0.158 (c_E_ = 302.96 mg/mL) and x_E_ = 0.105 (c_E_ = 191.06 mg/mL) for glyceline and ethaline, respectively.

Interestingly, this effect generally decreased with elevated temperature. For example, the 1:2 composition involving ethylene glycol was 53% more effective in terms of mole fraction solubility than the unimolar composition at 298.15 K but only 21% more efficient at 313.15 K. Finally, using ethylene glycol as a DES component resulted in better performance than utilizing glycerol. In the case of the optimal 1:2 composition, the former system was on average 1.34 times more efficient in terms of mole fractions than the latter one, although again this effect was more pronounced at 298.15 K than at 313.15 K. Thus, performed experiments proved that when using ETA or GLE a significant solubility advantage can be obtained with respect to water. Due to different temperature-related solubility profiles in water and DES this gain changes with increasing temperature. For the GLE, it ranged from 607 to 1553 times greater mole fraction solubility and for the system involving ethylene glycol the mole fraction solubility was from 911 to 1870 times larger if comparing 298.15 K and 313.15 K temperatures, respectively.

**Figure 1:**
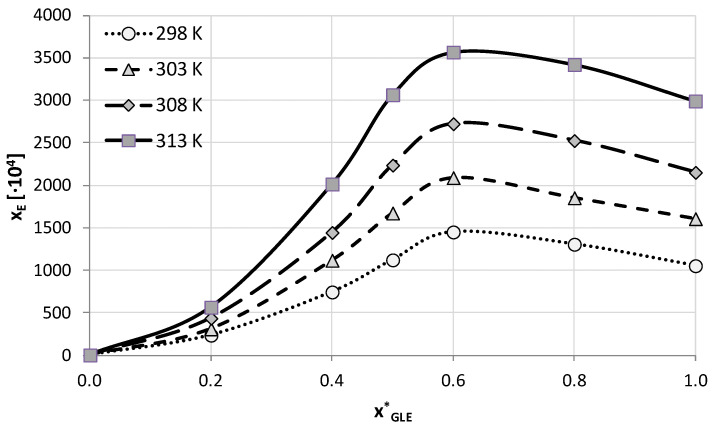
Mole fraction solubility of edaravone in mixtures of water and GLE (ChCl:GL = 1:2 molar ratio). On the abscissa, x*_GLE_ represents the mole fractions of the GLE in solute-free solutions. Detailed results are available in Table S1 in Supplementary Materials.

**Figure 2:**
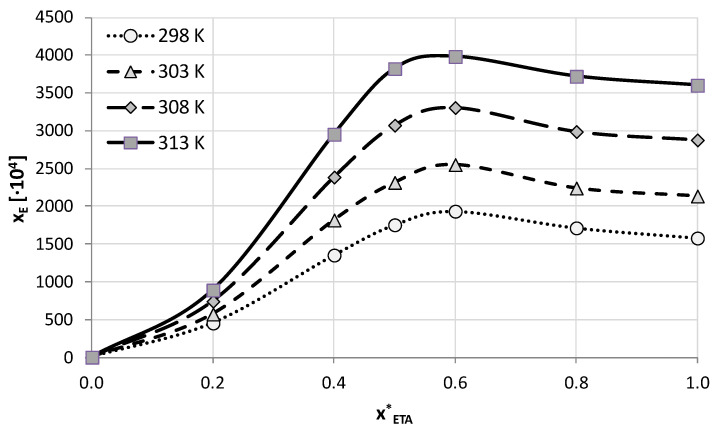
Mole fraction solubility of edaravone in mixtures of water and ETA (ChCl:EG = 1:2 molar ratio). On the abscissa, x*_ETA_ represents the mole fractions of ETA in solute-free solutions. Detailed results are available in Table S2 in Supplementary Materials.

There is an additional interesting aspect worth emphasizing. According to our earlier studies [53,54,55], the addition of water to the DES systems may improve the solubility of active pharmaceutical ingredients even more compared to neat eutectic systems. Therefore, the best-performing compositions for both studied DES systems were chosen for further experiments and used in mixtures with water in different proportions. A total of five compositions of DES-water binary solvents were studied, together with neat water and neat DES. Again, four different temperatures were studied as earlier. The obtained results of edaravone solubility measurements in these systems are collected in Figure 1 and Figure 2, and Tables S1 and S2 in Supplementary Materials for GLE and ETA, respectively.

The obtained solubility profiles show a very similar picture of edaravone dissolution for both studied here DES systems in binary mixtures with water. The addition of water to either ETA or GLE promotes edaravone dissolution compared to neat DES. For both systems the composition corresponding to the amount of DES in the solution equal x*_DES_ = 0.6 was found to be the most effective one. This aqueous mixture yielded solubility values at 298.15 K equal x_E_ = 0.193 (c_E_ = 459.69 mg/mL) and x_E_ = 0.145 (c_E_ = 344.22 mg/mL) for glyceline and ethaline, respectively.

The edaravone solubility Increase in the optimal binary composition was not very pronounced compared to neat DES, nonetheless it amounted to an average of 28% for glycerol-containing systems and 17% for systems with ethylene glycol. This effect however decreased when the temperature of measurements was elevated. Similarly, the general increase of edaravone solubility with increasing temperature was less pronounced in the case of the optimal composition than for the neat DES. When comparing the mole fraction solubility in the optimal binary composition with solubility in water an increase in the range from 836 to 1553 times and from 1116 to 2067 times is observed for glycerol and ethylene glycol, respectively.

As it was mentioned in the introduction, the solubility of edaravone was studied in a number of systems [12,13,14,15]. It is therefore interesting to compare the results obtained in this study with those available in the literature. Taking into account the best performing described in the literature [15], namely dichloromethane (x_E_ = 0.0688), the solubility of edaravone at 298.15 K is 1.53 times and 2.29 times larger in the case of GLE and ETA, respectively (optimal composition). When considering the best aqueous composition, this increase amounts to 2.11 times and 2.81 times, respectively, for GLE and ETA. Additionally, among a variety of binary solvents used for edaravone dissolution, none performed better than dichloromethane and therefore all are outperformed by the systems studied here. Such an important increase in solubility is a valuable result since it opens the possibility of using the studied systems in a potential new liquid form of the drug.

In order to assess the possibility of using the studied DES as practical solubilizers for edaravone, it is necessary to inspect the actual dosage of edaravone which is administered to patients in clinical practice. According to the literature [56,57], the recommended dosage is 60 mg administered daily. This means that the desired edaravone amount can be very easily achieved using the studied systems. In fact, taking into account the solubility at 298.15 K, such an amount can be found in 0.32 mL and 0.20 mL of neat DES, as well as in 0.18 mL and 0.14 mL of the optimal aqueous composition, for GLE and ETA, respectively. Of course, the tunable properties of both studied systems, in terms of molar composition and water content, could potentially be an important advantage in creating new liquid forms of edaravone.

### 2.2. Instrumental Characteristics of Saturated Systems

**Figure 3:**
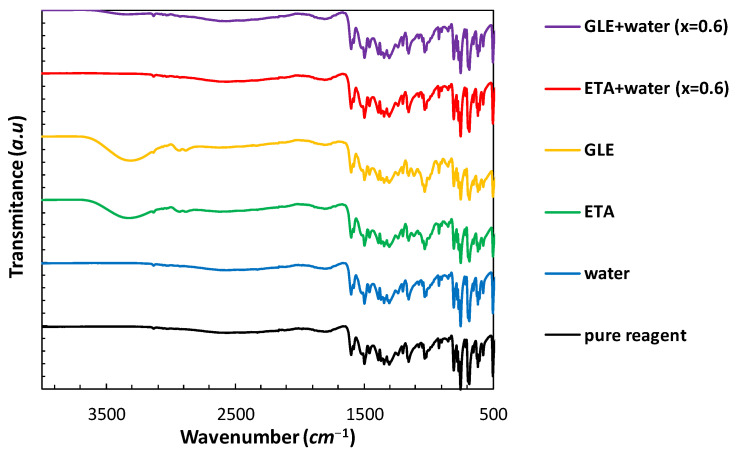
The FTIR spectra of edaravone precipitates after solubility measurements in neat DES, water and their mixtures (optimal composition). Values in parentheses indicate the mole fractions of DES in solute-free solutions. For comparison, the spectrum of pure edaravone is presented.

**Figure 4:**
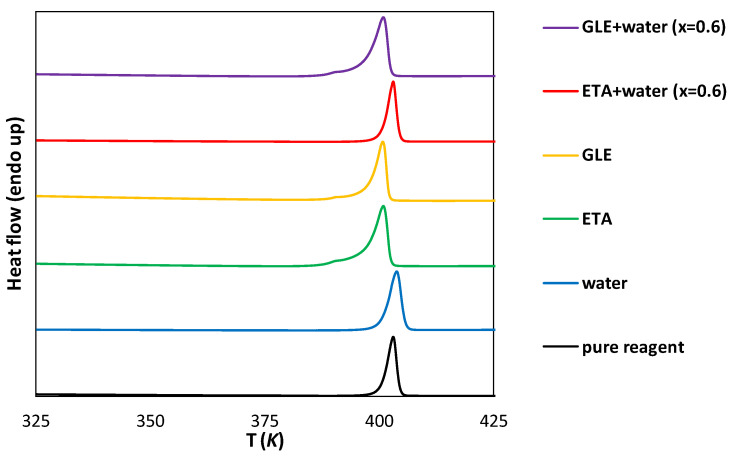
DSC curves of edaravone precipitates after solubility measurements in neat DES, water and their mixtures (optimal composition). Values in parentheses indicate the mole fractions of DES in solute-free solutions. The DSC curve of pure edaravone is also provided for comparison.

In order to evaluate the effect of the solvent on the solid residue, the analysis of the precipitates obtained after solubility measurements can be carried out. In the case of DES, such measurements might be difficult to perform due to the extremely low volatility of the solvent. This can make drying the samples difficult without the need for a high temperature or reduced pressure, which on the other hand could affect the crystalline form. Despite these difficulties, an attempt was made to characterize the solid residues collected after the flask-shake procedure by using IR spectroscopy and the DSC method. As it can be inferred from Figure 3 and Figure 4, in the solid state, edaravone does not form hydrates nor solvates, since both IR spectra and DSC thermograms of samples obtained after the solubility measurements in pure water are similar to the results obtained for the pure crystalline solute. Further, the melting points of the water sample and pure edaravone determined as onset values are almost identical, namely 400.98 K and 400.73 K, respectively. For comparison, the literature data range from 400.15 K to 403.15 K [13,14] was reported. In the case of ETA, GLE and GLE + water, the melting point decreased and characteristics for impurities were observed (T_m_ = 396.19 K, 397.99 K, 396.87 K, respectively). On the other hand, in the case of the ETA + water DSC thermogram, the lack of polymorphic and pseudo-polymorphic transitions can be observed. In this case, the onset value is also very close to the pure reagent (T_m_ = 400.84 K). In the case of IR spectra recorded for ETA and GLE, characteristic peaks of alcohols can be noticed. For instance, in the case of GLE two low-intense bands at 2880 and 2936 cm^−1^ (υ_as_CH_2_ and υ_s_CH_2_) and one wide band at 3343 cm^−1^ (υOH) can be observed, which are similar to data reported in the literature (2874, 2935, 3345 cm^−1^) [58]. The presence of the solvent shows that even after drying the samples under atmospheric pressure at room temperature for four weeks (which was applied in this study), pure edaravone is hardly recovered. Nevertheless, in all cases, the IR peaks (including the very low intensity ones such as 3125 cm^−1^) corresponding to edaravone can be identified and none of them are shifted, when compared to the pure reagent. This indicates the lack of structural changes, such as the formation of new hydrogen bonds with the solvent molecules in the solvate crystal.

### 2.3. Tautomersim of Edaravone

**Figure 5:**
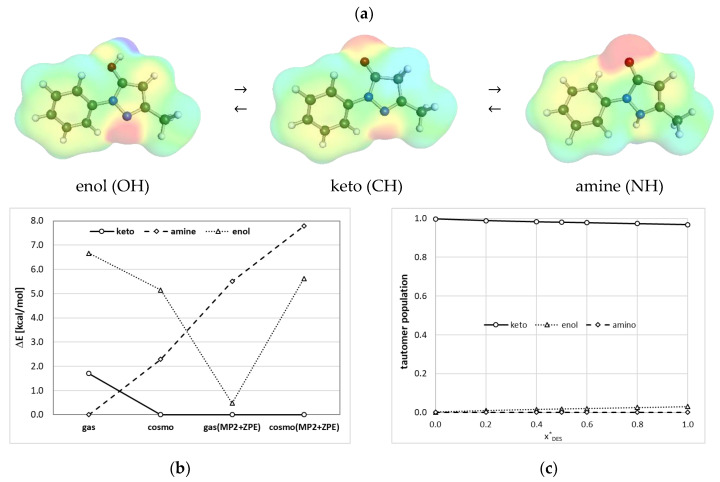
Tautomeric properties edaravone (**a**) tautomerisation scheme with charge densities distributions; (**b**) relative values of energy estimated in the gas and bulk phase using DFT approach and corrected for electron correlation and ZPE; (**c**) exemplary plot of tautomers’ populations in ETA (ChCl:EG = 1:2) at T = 298.15 K as a function of DES mole fraction (x*_DES_).

Before computations of any properties, the tautomeric equilibria of edaravone in studied systems are to be addressed. For this purpose, the hypothetical reactions of mutation of enol- or amine-forms into keto-tautomer were considered by computation of the values of the corresponding Gibbs free energy. Furthermore, the values obtained on the density functional theory (DFT) level (BP86/def2-TZVPD//BP97/def2-SVPD) were corrected for electron correlation and zero-point energy contributions (ZPE) using RI-MP2/def2-QZVPP//BP97/def2-SVPD and RI-BP97/def2-TZVPD//BP97/def2-SVPD levels, respectively. The obtained characteristics reveal interesting properties of edaravone, as documented in Figure 5. Considering results obtained from the DFT computations in the gas phase, it appears that the amine form is predominant, followed by keto and enol tautomers. This is a surprising conclusion, which is in the opposition to ^13^C-NMR experiments documenting that edaravone exists exclusively in the keto-form both in the gas phase and crystalline state [16]. Indeed, after the inclusion of electron correlation the order of tautomers in the gas phase changed drastically suggesting the predominance of keto-form followed by amine enol and ones. This strongly suggests the inadequacy of DTF for a proper description of tautomers’ order. On the other hand, if the bulk phase is characterized by values computed using the DFT-COSMO-RS approach, the keto-tautomer has been found to be the most stable form, which precedes amine and enol tautomers. The inclusion of the corrections coming from RI-MP2 and ZPE computations affects the energy values of edaravone tautomers but the sequence is unchanged. This implies that DFT is capable of adequate predication of the order of edaravone tautomers, but results of higher-level quantum chemistry should be included in quantitative analyses. In Figure 5c, there are provided plots exemplifying the tautomers population in ETA at room temperature as a function of the solute-free mole fraction of an aqueous mixture of this DES. It is clearly evident that the most dominant, and practically the only important, form of edaravone is the keto-isomer. These trends and the actual population values are almost insensitive to the way of computations and corrections originating from electron correlation and ZPE are negligible if the bulk condensed phase is considered. Therefore, all further computations done for edaravone characteristics in all studied systems included the keto-tautomer as the only contributor to the solute properties.

### 2.4. COSMO-RS Derived Edaravone Solubility in DES

**Figure 6:**
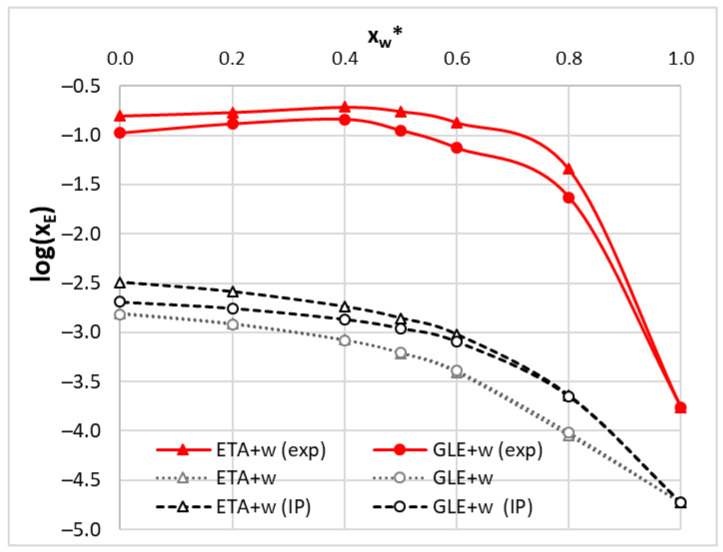
The room temperature trends of edaravone solubility in ethaline (ETA = ChCl:Et = 1:2) and glyceline (GLE = ChCl:Gl = 1:2) as a function of water molar fraction (x_w_*) in solute-free solutions. Solid red lines represent experimentally measured solubility, black dashed lines stand for solubility computed using the ion pair model and gray dotted lines characterize values computed according to the molecular model of DES.

Modeling of ionic liquids is usually done via the independent counter-ions approach. This molecular model is a straightforward one, which simply defines DES as neutral systems consisting of separated ions/species. In the case of ionic liquids based on choline chloride, both the cationic part and the chloride anion are treated as supermolecules, the structure of which is optimized as a neutral system. Hence, considered here DES consist of two neutral molecules, namely a hydrogen bond acceptor (ChCl) and a hydrogen bond donor (EG or GL). Alternatively, DES can be represented as ion pairs formed by direct interactions of its constituents. This results in ETA or GLE pairs stabilized by a net of hydrogen bonds between choline chloride and the polyol molecule. Since it is unknown which model is more suited as a representation of DES on the molecular level, both definitions were tested here. Hence, solubility computations using the COSMO-RS approach were performed twice. In the case of the molecular model, the proportions of solvent components, including water, were mimicked in the input files by preserving the experimental solvent mole fraction in the solute-free mixture. In the case of the ion pair model, the solvent mole fractions were adjusted, including changes imposed by ETA or GLE formation. The results of computed solubility at room temperature were exemplified in Figure 6 and confronted with experimentally measured data. It is clearly visible that COSMO-RS is able to properly represent the measured trend of solubility, but absolute values are very far from the experimental ones. The trends computed on both solvent models are fairly parallel to experimental ones, but the solubility is systematically underestimated. Unfortunately, the difference between computed and experimental solubility is not constant, which suggests that a simple shifting of computed solubility cannot help in increasing accuracy of computed solubility values. What is worth emphasizing, the application of a more complex model by inclusion of ETA or GLE pairs in the solubility computations leads to slightly better values of computed edaravone solubility. This conclusion consistently holds also for other temperatures and ChCl:polyol ratios.

### 2.5. Intermolecular Interactions in Eradavone-DES Systems

Deep eutectic solvents’ properties originate from specific mutual interactions of their constituents. For revealing these features, the structure and energetics of potential pairs were computed via an extensive conformational search leading to gaining the knowledge of the most stable intermolecular contacts comprising two or three molecules. Two classes of intermolecular contacts were presented and discussed below, which to the authors’ best knowledge are provided for the first time in the scientific literature. The characteristics encompass the formation and interactions of ion pairs in ETA or GLE, as well as edaravone dimerization and complexation with DES constituents.

#### 2.5.1. Mutual Affinity of DES Components

**Figure 7:**
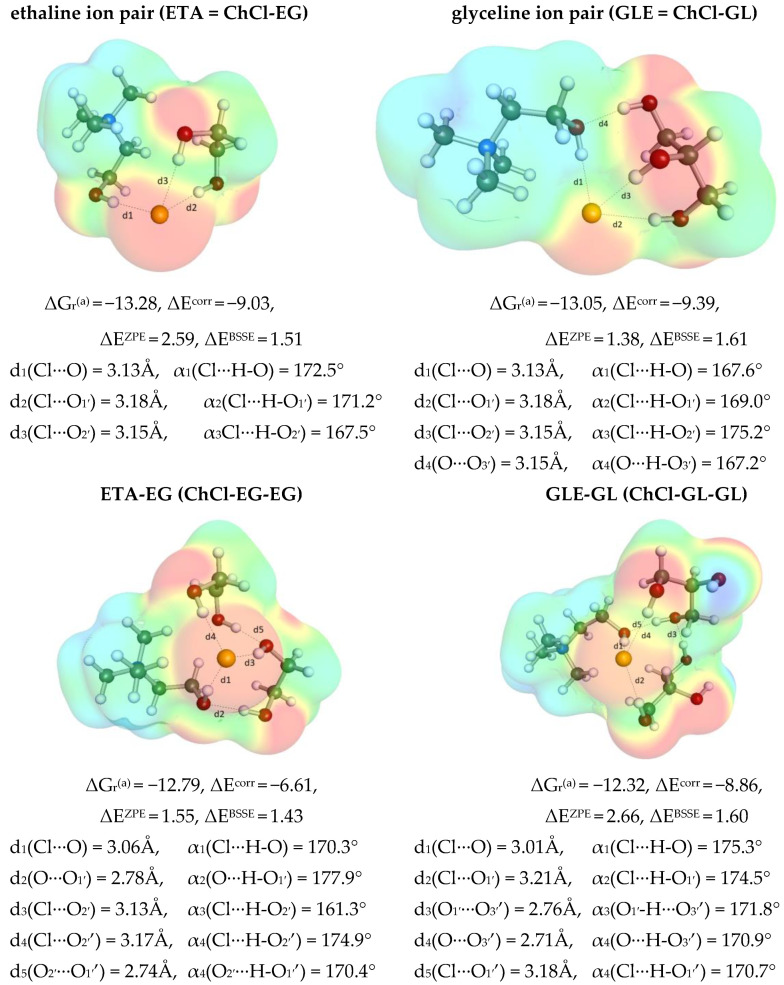
The most important geometric and energetic properties of intermolecular complexes identified in ETA and GLE. All thermodynamic contributions are expressed in kcal/mol and represent relative values for X + Y = XY reactions, ΔG_r_^(a)^ = the concentration-independent value of Gibbs free energy of reaction estimated in COSMOtherm using RI-BP86/def2-TZVPD//BP97/def2-SVPD, ΔE^corr^ = electron correlation computed at RI-MP2/def2-QZVPP//BP97/def2-SVPD level, ΔE^ZPE^ = zero vibrational contribution computed at RI-BP97/def2-TZVPD//BP97/def2-SVPD level and ΔE^BSSE^ = geometric based counterpoise correction BSSE DFT-C//BP97/def2-SVPD.

**Figure 8:**
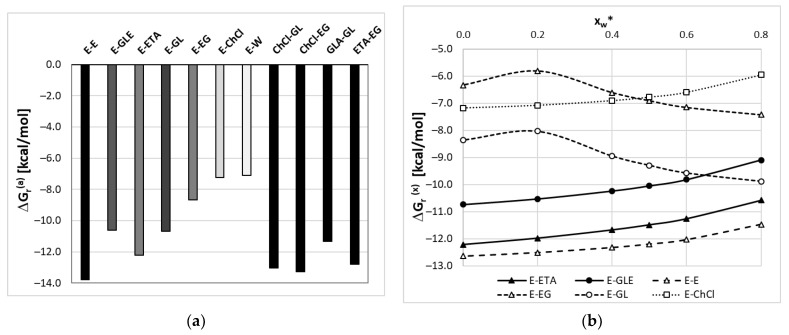
The values of edaravone affinity expressed as (**a**) activity-based Gibbs free energy (ΔG_r_^(a)^ = −RTln(K_a_); K_a_ = K_x_·K_γ_) and (**b**) concentration-dependent values of Gibbs free energies (ΔG_r_^(x)^(x) = −RTln(K_x_) in ethaline or glyceline (T = 298.15 K, ETA = ChCl:EG = 1:2, GLE = ChCl:GL = 1:2), where x_w_* denotes water mole fraction in solute-free solution. The following notation of intermolecular complexes was used: edaravone dimer (E-E), E pairs with glyceline ion pair (E-GLE), ethaline ion pair (E-ETA), glycerol (E-GL), ethylene glycol (E-EG), choline chloride (E-ChCl), and water (E-W).

The obtained collection provided in Figure 7 documents that ethylene glycol and glycerol can directly interact with choline chloride leading to ETA and GLE pairs, respectively. In both cases, the affinity is very strong, as suggested by the computed values of Gibbs free energies of ETA and GLE synthesis reaction, which are ΔG_r_(ETA) = −13.3 kcal/mol and ΔG_r_(GLE) = −13.1 kcal/mol, respectively, at 298.15 K. These interactions are among the strongest in the studied systems, as inferred from the comparison provided in Figure 8. It is interesting to notice that the origin of such a strong affinity of choline chloride is the ability to form a net of hydrogen bonds, the geometry features of which are documented in Figure 7. The colorful contours represent the distributions of electron charge densities and black lines indicate hydrogen bonding. According to the results of performed computations, the ETA pair is stabilized by three-center hydrogen bond (HB) of chloride with hydroxyl groups located either on choline or ethylene glycol. Both lengths and angle values of these contacts indicate strong hydrogen bonding. Glyceline pairs utilize the same geometric pattern, but an additional hydrogen bond is formed between the third hydroxyl group of glycerol with the oxygen of ChCl. In the cases of DES systems with other than unimolar proportion, the excess amounts of polyol molecules can interact with the above ion pairs. Although this leads to an increase in the number of hydrogen bonds in such intermolecular systems, the general geometric pattern of interaction ETA with EG or GLE with GL is preserved. Again, it is noticed that the chloride anion forms a three-centered hydrogen bond motif in both types of three-molecule clusters, but this time two different polyalcohol molecules are involved in such contacts. In addition, the intermolecular hydrogen bonds appear between either two ethylene glycol or glycerol molecules. Additionally, in the stabilization, the oxygen of choline acts as an acceptor in interactions with hydroxyl groups of EG or GL. The affinity of ETA or GLE pairs to the second polyalcohol molecule is still very high and is equal to ΔG_r_(ETA→ETA − EG) = −12.8 kcal/mol and ΔG_r_(GLE→GLE − GL) = −11.3 kcal/mol, respectively, at room temperature. Hence, it is rather expected that these clusters are the most representative structures of both ETA or GLY and are responsible for DES physicochemical properties.

#### 2.5.2. Edaravone Affinity to DES Components

**Figure 9:**
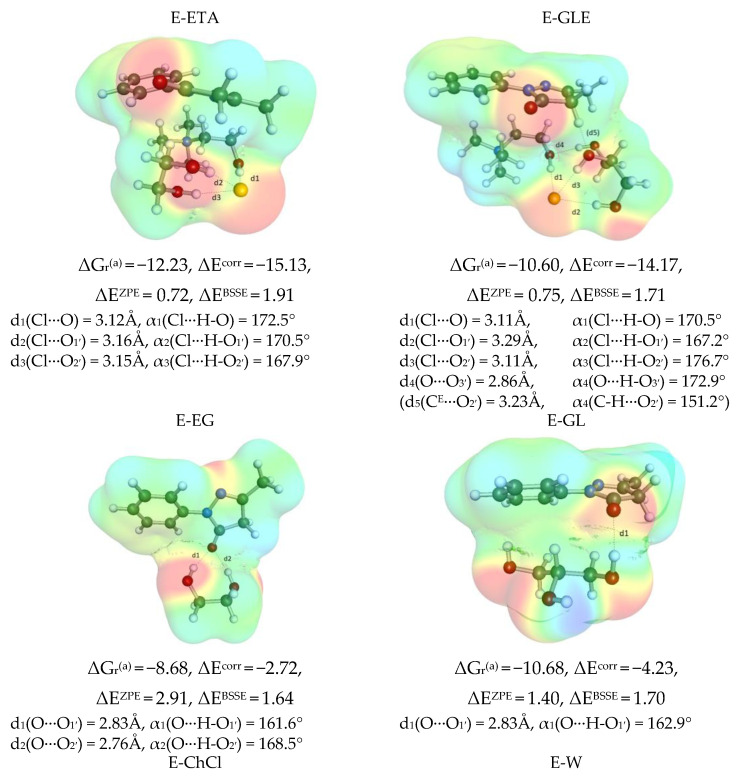

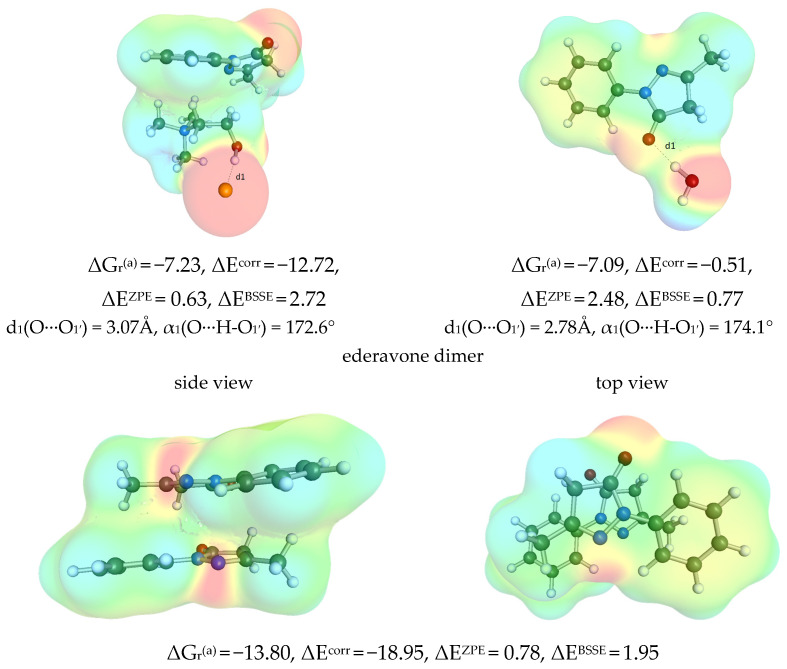
The most representative geometric and energetic properties of edaravone complexes with DES constituents. All thermodynamic contributions expressed in kcal/mol represent relative values for X + Y = XY reactions. Notation is the same as in Figure 7.

High solubility of edaravone in considered solvents is granted from the direct interactions occurring between constituents of DES. This is documented in Figure 8 by providing both activity and concentration-dependent values of the Gibbs free energy of reaction leading to intermolecular complexes involving edaravone. Among all considered complexes, the conformational analysis identified the edaravone dimers as the most stable complexes. This holds both for activity-based (ΔG_r_^(a)^) and concentration-dependent (ΔG_r_^(x)^) affinities. The latter slightly decreases with the dilution with water. The hetero-molecular complexes of edaravone with either ETA or GLE are also very stable and ethylene glycol has a higher affinity to edaravone compared to glycerol. This is true both for isolated EG or GL molecules and involved in ion pairs with ChCl. Similar influence of the solution composition is observed as for E-E dimers, as documented in Figure 8b. The most representative geometric and energetic properties of edaravone complexes with DES constituents are presented in Figure 9.

Since the keto-tautomer of edaravone has predominantly proton-accepting centers, it cannot form dimers stabilized by hydrogen bonds. However, there are strong stacking interactions between heterocyclic five-membered rings, which are responsible for strong attraction and E-E stabilization. This contact has been found as the most stabilizing among all other intermolecular clusters (ΔG_r_(2E→E-E) = −13.8 kcal/mol). In addition, edaravone shows high affinity to all DES constituents including water. This is related to the acceptance of hydrogen bonding of proton-donating molecules. In the case of ethylene glycol, two-centered hydrogen bonding is observed between both hydroxyl groups of EG. Pairs with glycerol or water are stabilized by a single HB. Finally, it is worth noting that the hydroxyl group of ChCl is involved in the interaction with chloride anion and edaravone is attracted via a similar electron density of heterocyclic nitrogen atoms of the non-polar region of a choline chloride molecule. The geometric pattern is responsible for the weak affinity, which is comparable to interactions of edaravone with a single water molecule. It is worth noting that the correction originating from electron correlation is very high in systems for which fragments are attracted by stacking interactions. On the contrary, if hydrogen boding dominates, the electron correlation correction turns out to be several times smaller.

### 2.6. Theoretical Model of Edaravone Solubility in DES

**Figure 10:**
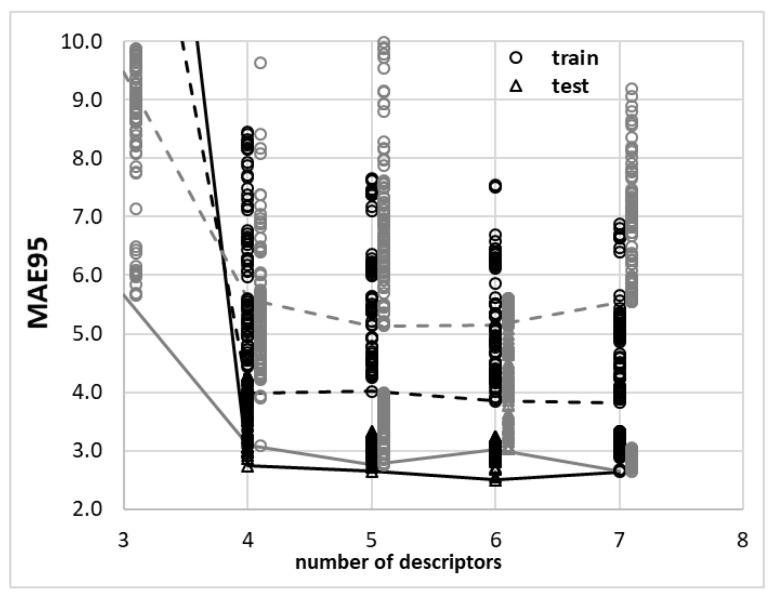
Representation of top 100 models for each number of the descriptors used in the MLR equation. Dotted and solid lines characterize the lowest values of MEA95 for train and test sets, respectively. Black ink was used for the presentation of models derived based on properties of edaravone in DES modeled as in ion pairs, while gray colors represent molecular models of either ETA or GLE.

The importance of augmenting experimental solubility reports with a theoretical model seems to be important for two main reasons. First of all, it might result in finding relationships between the properties of molecules and their observed dissolution profiles. This provides an opportunity to understand the molecular nature of the studied saturated systems. The second reason is of practical importance since it potentially enables finding solubility in systems not studied experimentally. Both aspects were kept in mind at this stage while aiming to develop a model of edaravone solubility in the studied ETA and GLE solvents. Since direct computations of edaravone solubility led to rather poor-quality prediction, an alternative approach is undertaken for the pool of data encompassing 72 measurements in a varying range of concentrations and temperatures. Bearing in mind that the number of observations is rather limited, the development of non-linear models formulated by the application of machine deep learning protocols seems to be not justified due to the possible risk of overfitting. This is due to the potentially large number of parameters introduced by such models. That is why, by following a small dataset quantitative structure–activity relationship (QSAR) modeling protocol [59], the interest was restricted to developing multiple linear regression models (MLRs). As can be found in the methodology part, the COSMO-RS-derived molecular descriptors were formulated and used for the purpose of edaravone solubility modeling. The use of such a type of descriptors is justified by the fact that they explicitly convey temperature and composition dependences of any system, including solutes in multicomponent solvents. In the initial phase, the analysis was focused on finding the optimal size of the linear regression equation. The results of the mean absolute error (MAE, 95%, test) values obtained for systematically generated models for a given number of descriptors defining the MLR equation are collected in Figure 10. This enables us to conclude that there are potentially many comparable models, which might be used for edaravone solubility back-computations based on subsets of 54 molecular descriptors obtained from COMO-RS computations.

**Figure 11:**
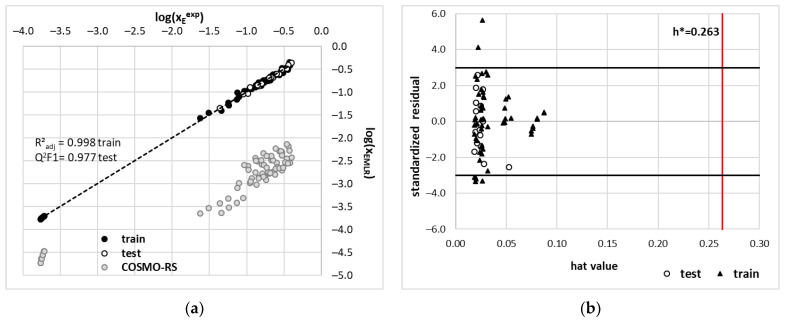
The results of the best MLR model applied for solubility computations of edaravone in studied DES. (**a**) computed solubility confronted with experimental values; (**b**) applicability domain characteristics of the model defined by Equation (1). The symbol h* defines the maximal hat values and is computed as follows h* = 3·(k − 1)/N, where k stands for the number of parameters (k = 6) and N is the number of data points in the training set (N = 57).

In Figure 10, the results of computations using either ion-pair or molecular model for descriptors computations are presented. It is visible that in general, the former model outperforms the latter and was used for the final model formulation. It seems that the optimal size of the MLR equation is equal to six considering the characteristics of the test set. Hence, among many thousands of potentially formulable MLR models for edaravone solubility description, the following one is selected as being balanced between size and accuracy:

(1) $$\begin{array}{cc} \log\left(x_{E}^{MLR}\right)= & 0.1722\left(\pm 0.0124\right)\cdot E_{E}^{tot}+0.5324\left(\pm 0.0125\right)\cdot E_{E}^{misfit}-1.2071\left(\pm 0.0252\right)\cdot \mu_{E}\\ & +0.7958\left(\pm 0.0156\right)\cdot pot2_{}^{inv}+0.3588\left(\pm 0.0104\right)\cdot \mathrm{HBA}+0.1363\left(\pm 0.0117\right)\cdot G_{r}^{AB3}\\ & 1.1069\left(\pm 0.0051\right) \end{array}$$

where the first two descriptors represent the total and misfit energy of edaravone in the mixture, the third descriptor, $\mu_{E},\;$quantifies the solute chemical potential, $pot2_{}^{inv}$ and HBA represent contributions from σ–potential and the last descriptor quantifies values of the Gibbs free energy of edaravone interactions with water. The application of Equation (1) leads to quite acceptable values of edaravone solubility back-computations, as documented in Figure 11. The following statistical measures describe the goodness of the fitting, namely internal validation parameters: R^2^_adj_ = 0.9987, Q^2^ = 0.9986, MAE(95%, train) = 0.0259, internal validation parameters: Q_2_^F1^(Test) = 0.9957, Q_2_^F2^(Test) = 0.9812, MAE(95% test) = 0.0304. All these measures suggest that the accuracy of the obtained model is acceptable. Additionally, the inspection of the applicability domain characteristics also confirms the reliability of the model defined in Equation (1). Only seven outliers were found out of 72 data points defined as predictions outside of tripled standard deviation. Nevertheless, the main limitation of the proposed approach is the narrow applicability, which is limited to edaravone. In general, local models are characterized by narrow applicability. Nevertheless, they can be used to describe the important structural and energetical features related to the solubility of a particular compound.

## 3. Materials and Methods

### 3.1. Solubility Measurements 

#### 3.1.1. Materials

Edaravone (E, CAS: 89-25-8, MW = 174.20 g/mol) purchased from Sigma Aldrich (Saint Louis, MO, USA) was the compound used during the solubility studies. Its purity was ≥99% according to the supplier. All of the NADES constituents were similarly delivered by Sigma Aldrich and included: choline chloride (ChCl, CAS: 67-48-1), glycerol (GL, CAS: 119 56-81-5) and ethylene glycol (EG, CAS: 107-21-1). Methanol (CAS: 67-56-1) was used as a solvent and was obtained from Avantor Performance Materials (Gliwice, Poland). The purity of all these compounds was ≥99% and they were used without initial procedures, except for choline chloride which was dried before use.

#### 3.1.2. Preparation of the Calibration Curve

The first step in obtaining a calibration curve was the preparation of a stock solution of edaravone with a concentration of 2.3 mg/mL. This initial solution was successfully diluted in 10 mL volumetric flasks with methanol and the obtained solutions with decreasing concentrations were measured spectrophotometrically. The wavelength corresponding to the highest absorbance was found to be 243 nm. Three separate curves were obtained, and the final curve resulted from averaging. The linear regression equation was found to be y = 85.603 + 0.0179 and the determination coefficient was equal R^2^ = 0.999. The limits of detection (LOD) and quantification (LOQ) were calculated as 5.899∙10^−4^ mg/mL and 1.770∙10^−3^ mg/mL, respectively.

#### 3.1.3. Preparation of Edaravone Samples in DES and in DES-Water Systems

All of the studied deep eutectic solvents included choline chloride, while the second constituent was either glycerol or ethylene glycol. In order to prepare a DES formulation, choline chloride was mixed with the second compound in different molar ratios in sealed test tubes and placed in a water bath at 363.15 K until the formation of a uniform solution. The obtained DES were either used in their neat form or water was added to them in different proportions in order to form binary systems.

During the next stage, edaravone was added in excess amounts to the test tubes containing both neat DES and binary mixtures, which enabled us to obtain its saturated solutions. Samples prepared in such a manner were placed in an Orbital Shaker Incubator ES-20/60 from Biosan (Riga, Latvia) and incubated for 24 h at different temperatures, namely 298.15 K, 303.15 K, 308.15 K and 313.15 K. The temperature adjustment accuracy was 0.1 K and its variability in a 24 h cycle was ±0.5 K. Apart from heating, all of the samples were mixed at 60 rev/min. The increased viscosity and density of DES systems required the samples to be centrifuged at 1000 rev/min for 5 min with the use of EBA 20 centrifuge from Hettich (Tuttlingen, Germany) enabling the undissolved precipitate to remain on the bottom of the test tubes. Next, the samples were filtered using a syringe equipped with a PTFE syringe filer with 0.22 µm pore size. In order to avoid precipitation, the test tubes, syringes and filters were initially heated at a temperature equal to the one of the handled samples. Finally, small amounts of the obtained filtrate were transferred to test tubes filled with methanol and the diluted samples were measured spectrophotometrically. Additionally, for the determination of the mole fractions of edaravone, the density of the samples was measured by weighing a fixed volume of the solution in 10 mL volumetric flasks.

#### 3.1.4. Edaravone Solubility Measurements

For determining the solubility of edaravone in the studied systems, the samples prepared, as described above, were measured spectrophotometrically using an A360 spectrophotometer from AOE Instruments (Shanghai, China). The spectra were recorded in the 190 nm to 500 nm wavelength range and 1 nm resolution. Methanol was used for the initial calibration of the spectrophotometer and for the dilution of the measured samples, which was conducted in order for the absorbance values to remain below 2.5. The absorbance values at 243 nm were taken into account and based on the calibration curve prepared earlier, the concentration of edaravone in the samples was determined, followed by its mole fractions. The values were averaged from three separate measurements.

### 3.2. DSC and FTIR Measurements

The solid residues remaining in the test tubes after solubility measurements were dried on air and analyzed by Fourier transform infrared spectroscopy (FTIR) and differential scanning calorimetry (DSC). The FTIR spectra were recorded using a Spectrum Two spectrophotometer from Perkin Elmer (Waltham, MA, USA) equipped with an attenuated total reflection (ATR) device. The samples were analyzed in a 450–4000 cm^−1^ wavenumber range. For the DSC measurements, a DSC 6000 calorimeter from PerkinElmer (Waltham, MA, USA) was used. The heating rate was set to 10 K/min and the inert atmosphere was provided by nitrogen with a 20 mL/min flow. The samples were placed in standard aluminum pans and the apparatus was initially calibrated using indium and zinc standards.

### 3.3. QSPR Modeling

Modeling of the edaravone solubility in studied DES was performed by the development of multiple linear regression (MLR) models using molecular descriptors computed in COSMOtherm utilizing COSMO-RS theory. Since solubility computations in this theoretical framework provide many details in the generated output files, they were used for molecular descriptor collection without any extra computation costs. Since there were 55 collected parameters, the MLR might be developed by a full exploration of the whole descriptors hyperspace and all possible combinations of descriptors were considered. The molecular descriptors were standardized by subtracting the mean values and dividing them by the standard deviation. Then, all interrelated parameters (R^2^ > 0.5) were omitted. Dataset was split into training (80%) and test sets (20%) using Kennard-Stone based method [60]. The best set of parameters was selected by a systematic search of all combinations of independent descriptors. The quality of predictions was evaluated using all the important internal (R^2^_adj_, Q^2^_LOO_, MEA(95% train) and external (Q^2^_F1_, Q^2^_F2_, MEA(95% test)) validation metrics. All the above steps were performed using the freeware version of QSAR model development tools provided by DTC Lab. Software (http://teqip.jdvu.ac.in/QSAR_Tools/(accessed on 10 December 2022)) [59,61]. The model formulation was completed with applicability domain [62] analysis.

### 3.4. Molecular Descriptors Computations

For effective MLR models formulation, the set of 55 molecular descriptors was formulated utilizing various molecular properties computed using the COMSO-RS approach implemented in COSMOtherm. 

#### 3.4.1. Cosmo-RS Derived Solubility

The COSMO-RS (Conductor-like Screening Model for Real Solvents) [63,64,65,66] was applied for the theoretical characteristics of solid–liquid multi-component systems. This commonly used framework takes advantage of the first principle quantum chemistry computations augmented with statistical thermodynamics for assessments of thermodynamic properties including chemical activities. Although it was designed for liquid systems, the solid–liquid equilibria (SLE) can also be treated, if only fusion data are provided either from direct measurements or external computations. In the case of edaravone, both the melting temperature, T_m_ = 403.15 K, and heat of fusion H_fus_ = 29.91 kJ/mol are known [14] and as such were used for solubility computations. The fusion data are indispensable since the values of the chemical potential of the solute in the saturated conditions are determined by the activity of the pure solid phase, according to the fundamental formula:

(2) $$\ln\left(a_{i}^{s}\right)=\ln\left(\gamma_{i}^{\mathrm{sat}}x_{i}^{\mathrm{sat}}\right)=-\frac{\Delta_{\mathrm{fus}}G_{i}^{m}\left(p,T\right)}{\mathrm{RT}}$$

where $\Delta_{\mathrm{fus}}G_{i}^{m}\left(p,T\right)$ is the partial molar Gibbs free energy of fusion at the solubility measurement conditions. Practically, solubility is commutated by iteratively solving the following equation:

(3) $$ln\left(\gamma_{i}^{sat,i+1}x_{i}^{sat,i+1}\right)=\frac{1}{RT}\left(\mu_{i}^{o,liq}-\mu_{i}^{\left(i\right)}\left(\gamma_{i}^{sat,i}x_{i}^{sat,i}\right)+max\left(0,\Delta_{fus}G_{i}^{m}\left(p,T\right)\right)\right)$$

In the above equation superscripts *i* and *i + 1* denote the values obtained in two subsequent iterations. The iterative cycle is repeated until convergence is achieved, which means that the computation is supposed to be interrupted if the difference in the computed solubility drops below a defined threshold value. The bulk phase used for solubility computations has the same composition as the solute-free solvent used in experimental measurements.

#### 3.4.2. Intermolecular Interactions Characteristics

The values of solute-solvent affinities were estimated similarly as already reported in our previous studies [67,68,69] and here only brief remarks are provided. The affinity represents the values of Gibbs free energies of reaction X + Y = XY, where X and Y stand for either edaravone or DES constituents. Particularly, in the case of edaravone (E) dimerization, X = Y = E and XY = E-E. Similarly, for the ion pairs, X is set to choline chloride (ChCL) and Y stands for either glycerol (GL) or ethylene glycol (EG) which resulted in XY equal to ethaline (ETA) or glyceline (GLE). Furthermore, the interactions of ion pairs were described assuming X = ETA or GLE and Y was set to one of the following species: E, EG or GL leading to clusters comprising three molecules. The affinities of edaravone to DES were computed for reactions in which X = E and Y was equal to one of DES constituents, namely, Y = either ETA, GLE, EG, GL, ChCl or water (W). The values of Gibbs free energy were expressed either as activity-based Gibbs free energy (ΔG_r_^(a)^ = −RTln(K_a_); K_a_ = K_x_·K_γ_) or concentration-dependent values of Gibbs free energies (ΔG_r_^(x)^ = −RTln(K_x_). The following notation of intermolecular complexes was used: edaravone dimer (E-E), E pairs with glyceline ion pair (E-GLE), ethaline ion pair (E-ETA), glycerol (E-GL), ethylene glycol (E-EG), choline chloride (E-ChCl), and water (E-W).

#### 3.4.3. Affinity Computations

Computations of the affinity were performed using a thermodynamic cycle scheme, which relies on geometry optimization both in the gas and bulk phases followed by thermal free energy contribution determined via the COSMO-RS approach. Since thermodynamic characteristics of the reaction in the gas phase is critical for the final accuracy [70], quantum chemistry computations were performed for the assessment of electron correlation corrections $\Delta E_{}^{\mathrm{cor}},$ and zero point vibrational energy $\Delta E_{}^{\mathrm{ZPE}}$. The first contribution was computed on an RI-MP2 level of theory and was included using an extended Ahlrichs def2-QZVPP basis set [71] utilizing the geometry determined in the previous step, RI-MP2/def2-QZVPP//BP97/def2-SVPD. In addition, the values of the zero point vibrational energy (ZPE) were computed at RI-BP97/def2-TZVPD//BP97/def2-SVPD level. The BSSE can be estimated using DFT-C approach, which formulation includes atom-atom many-body corrections and is a parameterized geometry-based method [72]. In general, counterpoise correction can be time consuming, but this geometry-based approach is very efficient since it utilizes only information about molecular geometries. However, the BSSE estimated via DFT-C was parametrized for BP97/def2-SVPD optimizations and this level was assumed as default both for conformational analysis of monomers and contacts.

#### 3.4.4. Intermolecular Interactions as Molecular Descriptors

Solubility computations provide very detailed information about the energetic contributions to the system’s stability. This enables to obtain, without any additional computations, a set of descriptors utilizing the Coulomb interactions (termed as “misfit” energy), hydrogen bonding and the Van der Waals (vdW) interactions occurring between surface segments. Such energetic information is system-dependent and adopt values according to external conditions and the system composition. Hence, the absolute values of these contributions as well as the total energy of the given system were used as molecular descriptors. In addition, the relative values of contributions to the interaction energies were used. These energetic molecular descriptors were computed for *i*-th components as follows:

(4) $$\Delta E_{j}^{i}=\frac{E_{j}^{i}}{\sum_{j}E_{j}^{i}}$$

where *j* stands for misfit, HB or VdW contributions. 

#### 3.4.5. Cosmo-RS Derived σ-Potentials as Molecular Descriptors

**Figure 12:**
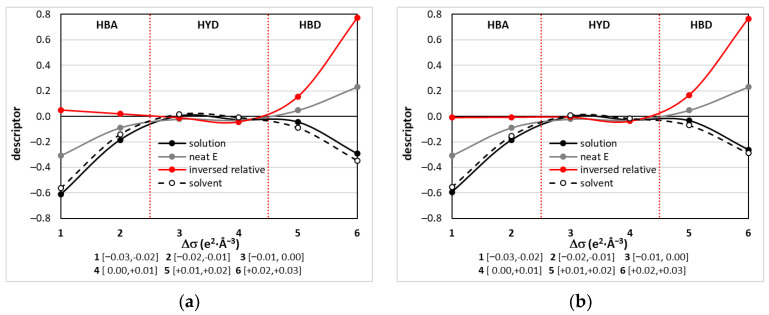
Illustration of σ-potential-related molecular descriptors characterizing systems with the highest edaravone solubility, which are (**a**) ETA and (**b**) GLE with x*_DES_ = 0.6 at T = 298.15 K. The grey lines represent properties of neat edaravone while black solid and dashed lines characterise solute-free solutions and the mixture, respectively. Red line denoted as the inverse relative curve represents values between inversed plot of neat edaravone and solute-free solvent.

It seems to be preferable to use such physicochemical properties, which are directly related to chemical structure in multi-component systems at defined external conditions. Fortunately, the COMOS-RS framework offers a very intuitive set of chemical systems characteristics that might be used for DML development. For example, [73], the σ-chemical potential trends, μ_s_(σ), are rich in information characterizing the chemical diversity of a given compound in varying environments. It represents the summarized σ-profiles [74] which are simply histograms of charge density distributions of given compounds in the mixture. It is argued [74] that the electronegative charge distribution in the range σ ∈ <−0.03, −0.01> characterizes the affinity for HB donors, HBA (hydrogen bonding acceptability). On the opposite scale, there is an electropositive polarity interval σ ∈ <0.01, 0.03> to which the affinity for HB acceptors is associated, HBD (hydrogen bond donicity). The intermediate range, σ ∈ <−0.01, +0.01>, characterizes contributions to non-polar interactions and is regarded as a measure of hydrophobicity, HYD. For practical purposes, the σ-chemical potential, typically provided with 61 points resolution, undergoes data reduction by averaging over 0.01 *e/Å^3^* range, which leads to six desecrators, namely spot1 = µ(σ ∈ <−0.03, −0.02), spot2 = µ(σ ∈ <−0.02, −0.01), spot3 = µ(σ ∈ <−0.01, 0.00), spot4 = µ(σ ∈ <0.00, +0.01), spot5 = µ(σ ∈ <+0.01, +0.02), and spot6 = µ(σ ∈ <+0.02, +0.03>. These descriptors can be computed in such a manner for any mixture, free solute or solute-free solvent with a given composition. This was illustrated in Figure 12 for systems characterized by the highest edaravone solubility at room temperature, as proven by performed measurements. Both ETA and GLE have very similar profiles showing activities both as hydrogen bond donors and acceptors. 

### 3.5. Calculation Details

Theoretical characteristics of analyzed compounds started with conformational analysis for an adequate representation of structural diversity. The initial structures of monomers were taken from the public PubChem database [75] and processed using the BIOVIA COSMOconf 2020 program [76] dedicated to generating the most energetically favorable conformations. The algorithm performs a series of optimizations and the reduction of a number of structures, leading to the most probable conformers. The working part utilizes BIOVIA TURBOMOLE 2021 (release V7.5.1) [77] for geometry optimizations. 

The multicomponent clusters were also prone to an extended conformational analysis done by molecular surface segments statistics invoked in the COSMOtherm program via the command “CONTACT = {1 2} ssc_probability ssc_weak ssc_ang = 15.0”. Many automatically generated structures were further optimized and clustered on the same level as monomers. Only clusters with stabilisation energy within a 5 kcal/mol window were included in the final set of conformers. Hence, the intermolecular interaction energies in the gas phase correspond to the results of full geometry optimization of both monomers and pairs using RI-DFT with BP97 GGA functional using def2-SVPD basis set and Grimme D3-BJ dispersion corrections [78]. This functional was used for multicomponent clusters optimization instead of the default BP86 density functional since it is more suited for multicomponent systems [72]. However, the final values of the affinities were obtained using the most sophisticated parametrization available in BIOVIA COSMOtherm 2021 [76,78]. However, the application of BIOVIA COSMOtherm 2021 [76] for equilibrium constants computations requires selection none of available parametrizations, which in the case of this work corresponded to the BP_TZVPD_FINE_21.ctd set. 

## 4. Conclusions

The solubility of edaravone was determined in designed solvents by optimizing their composition. Experimentally proved optimal molar ratio of choline chloride to used polyols was found to be 1:2, irrespective of the temperature. Among two studied deep eutectic solvents (DES), ethaline (ETA) and glyceline (GLE), the former can be regarded as a better solubilizing agent leading to 911 times higher solubility compared to neat water at room temperature. The solubility can be further increased by about 26% if water is added to neat DES. Hence, the optimal solvent comprised x*_ETA_ = 0.6. A similar conclusion is also held for GLE. This confirms the phenomenon previously observed for other active pharmaceutical ingredients that dilution of DES or NADES with water promotes their dissolution. The studied systems outperform the solvents discussed in the literature to date. Additionally, the recommended dosage of edaravone administered to patients, i.e., 60 mg daily, can be easily achieved with both studied DES. This beneficial solvation of edaravone, together with the possibility of fine-tuning the considered systems, opens the possibility of creating new liquid forms of this drug. 

Another interesting pattern was confirmed in relation to the optimal selection of the hydrogen bond donor in the choline chloride eutectic mixtures. The lower the number of hydroxyl groups, the higher solubility is observed for many drug-like solutes.

The computed intermolecular interactions help understand the microstructure of the studied systems. Here, two- and three-molecular clusters were optimized and used for affinity computations. The most stable pair was found for edaravone dimer stabilized by stacking interactions between five-membered heterocyclic rings. These interactions are stronger even than the ones between neat DES constituents. In addition, edaravone exhibits a very strong affinity to ion pairs, ETA and GLE, which are stronger than interactions with ethylene glycol or glycerol. The slightly higher solubility of edaravone in the former DES can be attributed to a stronger interaction with ETA compared to GLE.

Finally, it was possible to formulate a linear regression model for solubility computations in the studied systems. The acceptably good accuracy, described both by provided internal and external statistical measures, allows for screening new DES, which will share both high solubility effectiveness and low negative environmental impact. The provided details of the applicability domain can effectively guide the selection of new candidates for further experiments and the design of new eutectic solvents.

## Figures and Tables

**Table 1:** The solubility of edaravone dissolved in DES in different molar ratios (choline chloride first) at various temperatures. Values expressed as mole fractions (x_E_, 10^4^) and concentrations (c_E_, mg/mL). Standard deviation values are given in parentheses.

T (K)	1:1 Molar Ratio		1:2 Molar Ratio		1:4 Molar Ratio	
	x_E_ (∙10^4^)	c_E_ (mg/mL)	x_E_ (∙10^4^)	c_E_ (mg/mL)	x_E_ (∙10^4^)	c_E_ (mg/mL)
choline chloride + glycerol (GLE)						
298.15	786.83 (±3.34)	138.18 (±1.21)	1052.85 (±68.85)	191.06 (±11.91)	926.44 (±23.62)	177.17 (±4.09)
303.15	1173.65 (±39.25)	202.99 (±6.76)	1606.43 (±50.52)	282.25 (±8.03)	1321.28 (±36.17)	246.26 (±5.86)
308.15	1615.31 (±40.69)	274.07 (±6.88)	2151.57 (±64.56)	369.09 (±11.3)	1861.97 (±40.45)	336.43 (±6.12)
313.15	2429.30 (±72.71)	398.02 (±10.92)	2991.42 (±64.1)	492.12 (±9.58)	2739.72 (±65.05)	470.20 (±9.06)
choline chloride + ethylene glycol (ETA)						
298.15	1031.93 (±62.91)	189.66 (±10.5)	1579.47 (±70.73)	302.96 (±11.01)	1306.68 (±55.75)	280.14 (±10.35)
303.15	1400.98 (±40.74)	251.75 (±7.12)	2135.68 (±42.46)	392.28 (±6.53)	1677.66 (±47.52)	346.64 (±7.87)
308.15	2089.82 (±49.36)	359.76 (±7.92)	2878.22 (±133.85)	499.02 (±18.34)	2434.99 (±114.12)	467.15 (±16.58)
313.15	2966.24 (±120.59)	484.49 (±16.00)	3602.21 (±117.41)	592.85 (±14.78)	3428.20 (±105.69)	601.77 (±12.46)

## Data Availability

Not applicable.

## Supplementary Materials

The following supporting information can be downloaded at: https://www.mdpi.com/article/10.3390/molecules28020629/s1, Table S1. The solubility of edaravone dissolved in mixtures of water and DES comprising choline chloride and glycerol in 1:2 molar ratio (GLE) at various temperatures. Values expressed as mole fractions (x_E_, ∙10^4^) and concentrations (c_E_, mg/mL). Standard deviation values are given in parentheses. Table S2. The solubility of edaravone dissolved in mixtures of water and DES comprising choline chloride and ethylene glycol in 1:2 molar ratio (ETA) at various temperatures. Values expressed as mole fractions (x_E_, 10^4^) and concentrations (c_E_, mg/mL). Standard deviation values are given in parentheses.
