# Supplementary material for: Intermolecular Interactions of Edaravone in Aqueous Solutions of Ethaline and Glyceline Inferred from Experiments and Quantum Chemistry Computations
Source: Molecules. 2023 Jan 7;28(2):629. doi: 10.3390/molecules28020629 (PMC9863297; doi:10.3390/molecules28020629)
Supplement: Supplementary file 1 [file molecules-28-00629-s001.zip › molecules-2121467-supplementary.pdf]

Supplementary Materials for Intermolecular interactions of Edaravone in aqueous solutions of Ethaline and Glyceline inferred from experiments and quantum chemistry computations.

Piotr Cysewski [1]*, Tomasz Jeliński [1], Maciej Przybyłek [1]

[1] Department of Physical Chemistry, Pharmacy Faculty, Collegium Medicum of Bydgoszcz, Nicolaus Copernicus University in Toruń, Kurpińskiego 5, 85-096 Bydgoszcz, Poland
* Correspondence: Piotr.Cysewski@cm.umk.pl

Table S1. The solubility of edaravone dissolved in mixtures of water and DES comprising choline chloride and glycerol in 1:2 molar ratio (GLE) at various temperatures. Values expressed as mole fractions (x_E, $\cdot10^4$) and concentrations (c_E, *mg/ml*). Standard deviation values are given in parentheses.

x^*_{GLE}	**298.15 *K***		**303.15 *K***		**308.15 *K***		**313.15 *K***	
	x_E ($\cdot10^4$)	c_E (*mg/ml*)	x_E ($\cdot10^4$)	c_E (*mg/ml*)	x_E ($\cdot10^4$)	c_E (*mg/ml*)	x_E ($\cdot10^4$)	c_E (*mg/ml*)
**0.0**	1.73(±0.01)	1.72(±0.01)	1.78(±0.01)	1.79(±0.02)	1.84(±0.02)	1.87(±0.01)	1.93(±0.01)	1.96(±0.01)
**0.2**	235.51(±9.67)	115.11(±4.41)	309.2(±4.30)	148.29(±1.98)	435.07(±4.04)	201.28(±1.21)	564.61(±7.05)	251.54(±2.67)
**0.4**	743.88(±5.92)	233.13(±1.43)	1116.64(±2.70)	328.49(±0.84)	1444.07(±14.86)	402.14(±2.73)	2014.09(±17.34)	513.56(±2.32)
**0.5**	1121.47(±10.85)	298.98(±3.19)	1673.69(±20.61)	414.6(±3.61)	2236.41(±28.00)	517.95(±3.12)	3065.17(±55.82)	646.93(±6.00)
**0.6**	1450.22(±11.26)	344.22(±2.54)	2089.77(±58.10)	464.06(±10.07)	2723.73(±61.99)	567.03(±8.81)	3567.08(±42.51)	691.57(±4.95)
**0.8**	1305.70(±26.15)	265.81(±4.17)	1852.98(±39.43)	363.68(±5.58)	2525.56(±24.65)	471.98(±3.89)	3420.95(±31.16)	602.41(±4.73)
**1.0**	1052.85(±68.85)	191.06(±11.91)	1606.43(±50.52)	282.25(±8.03)	2151.57(±64.56)	369.09(±11.3)	2991.42(±64.10)	492.12(±9.58)

Table S2. The solubility of edaravone dissolved in mixtures of water and DES comprising choline chloride and ethylene glycol in 1:2 molar ratio (ETA) at various temperatures. Values expressed as mole fractions (x_E, $\cdot10^4$) and concentrations (c_E, *mg/ml*). Standard deviation values are given in parentheses.

x^*_{ETA}	**298.15 *K***		**303.15 *K***		**308.15 *K***		**313.15 *K***	
	x_E ($\cdot10^4$)	c_E (*mg/ml*)	x_E ($\cdot10^4$)	c_E (*mg/ml*)	x_E ($\cdot10^4$)	c_E (*mg/ml*)	x_E ($\cdot10^4$)	c_E (*mg/ml*)
**0.0**	1.73(±0.01)	1.72(±0.01)	1.78(±0.01)	1.79(±0.02)	1.84(±0.02)	1.87(±0.01)	1.93(±0.01)	1.96(±0.01)
**0.2**	454.10(±5.48)	220.96(±2.27)	574.18(±8.89)	269.62(±3.62)	742.26(±18.45)	331.05(±6.32)	890.33(±10.84)	381.65(±3.10)
**0.4**	1353.93(±5.00)	406.66(±1.15)	1819.02(±41.45)	505.10(±7.17)	2386.29(±27.09)	602.61(±3.89)	2954.32(±49.04)	683.73(±5.94)
**0.5**	1754.56(±31.02)	456.55(±6.04)	2316.25(±38.27)	554.20(±5.35)	3073.71(±53.04)	663.59(±6.82)	3822.75(±61.93)	743.62(±7.61)
**0.6**	1934.93(±34.18)	459.69(±5.59)	2552.36(±52.56)	560.61(±8.87)	3306.79(±48.29)	670.27(±6.19)	3982.72(±70.26)	752.91(±6.66)
**0.8**	1713.96(±31.15)	363.37(±5.15)	2241.64(±42.78)	451.55(±6.38)	2989.20(±42.33)	561.30(±6.00)	3720.80(±28.54)	655.40(±3.72)
**1.0**	1579.47(±70.73)	302.96(±11.01)	2135.68(±42.46)	392.28(±6.53)	2878.22(±133.85)	499.02(±18.34)	3602.21(±117.41)	592.85(±14.78)
